# Influence of ShuJinHuoXue Tablets on Ischemia Reperfusion Injury of Animals’ Skeletal Muscle

**DOI:** 10.3390/molecules17088494

**Published:** 2012-07-16

**Authors:** Zhihong Tong, Fang Yu, Zhonghua Liu, Haidong Liang

**Affiliations:** 1 Hands and Feet Microsurgery, Dalian Municipal Central Hospital, Dalian 116033, China; 2 School of Medicine, Dalian University, Dalian 116033, China; 3 Orthopedic Department, Changchun University of Traditional Chinese Medicine Affiliated Hospital, Changchun 130021, China

**Keywords:** ShuJinHuoXue tablets, CK, SOD, antioxidant, GSH-Px

## Abstract

Ischemia-reperfusion (IR) can lead to serious tissue oxidative injury in animals. ShuJinHuoXue tablet (SJHXT) is a Chinese Traditional Medicine which can relax the muscles and stimulate the blood circulation and has been used as a clinical medicine. In the present study, we investigated the effects of SJHXT pretreatment on oxidative injury using an animal model of acute limb IR. Results showed that SJHXT pre-treatment (200, 300 and 400 mg/kg/day) markedly reduced serum endothelin-1 (ET-1), thromboxane B2 (TXB_2_) levels and thromboxane B2/6-keto- prostaglandin F1α (TXB_2_/6-Keto-PGF_1α_), wet weight/dried weight (W/D) ratio, myeloperoxidase (MPO), creatine kinase (CK), lactate dehydrogenase (LDH) activities, and increased serum nitric oxide (NO), 6-Keto-PGF_1α_ levels and NO/ET-1 ratio in the IR+SJHXT groups. In addition, the SJHXT pre-treatment (200, 300 and 400 mg/kg/day) markedly reduced skeletal muscle Ca^2+^, malondialdehyde (MDA) levels, increased Na^+^-K^+^-ATPase, Ca^2+^-Mg^2+^-ATPase, superoxide dismutase (SOD), catalase (CAT), and glutathione peroxidase (GSH-Px) activities. Our results suggest that SJHXT pre-treatment may improve skeletal muscle blood vessel microcirculation, decrease skeletal muscle oxidative injury and enhance antioxidant enzymes activities in IR animals.

## 1. Introduction

Ischemia-reperfusion injury (IRI) is defined as the paradoxical exacerbation of cellular dysfunction and death following the restoration of blood flow to previously ischemic tissues. Reestablishment of blood flow is essential to salvage ischemic tissues, however reperfusion itself paradoxically causes further damage to the ischemic tissue, threatening function and viability of the organ. Reactive oxygen (ROS) and nitrogen species (RNS) that have been implicated in tissue IRI include the hydroxyl radical (OH-), hydrogen peroxide (H_2_O_2_), superoxide anion O_2_, nitric oxide (NO^−^), and peroxynitrite (ONOO^−^) [1,2]. These reactive species may arise very early during IRI, coming from several sources such as the electron transport chain in mitochondria, the xanthine/xanthine oxidase reaction [3,4] and RNS from cNOS (NO^−^, ONOO^−^) [5,6]. The role of NO in ischemia/reperfusion injury remains controversial in that NO shows both cytoprotective and cytotoxic actions [7]. Superoxide dismutase enzyme (SOD) is a potent protective enzyme that can selectively scavenge O_2_^−^ by catalyzing its dismutation to H_2_O_2_ and oxygen (O_2_). The other antioxidant enzyme, catalase (CAT), catalyzes the conversion of H_2_O_2_ to water and oxygen. Assessment of the activities of particular free radical scavenging enzymes and lipid peroxidation end-product levels in plasma and/or muscle tissue would enhance our understanding of the mechanism in muscle ischemia-reperfusion injury.

Many studies have implicated oxygen-derived molecules (free radicals) as the mediators of reperfusion injury in a variety of tissues, including skeletal muscle [8,9,10,11,12,13]. IRI in skeletal muscle, particularly in the lower limbs, is a frequent clinical problem following the surgical repair of abdominal aortic aneurysms as well as traumatic crush injuries, massive hemorrhages, vascular stenosis, thromboembolic events, organ transplantation, and cardiovascular surgery [14,15]. Ischemia and the subsequent reperfusion of the skeletal muscle tissue stimulate an inflammatory response in the affected muscles, as well as induce injury to other tissues. In severe cases of limb ischemia, the resulting reperfusion is associated with high mortality resulting from multiple system organ failure [16,17].

ShuJinHuoXue tablets are a Chinese medicine which is produced according to a herbal prescription. It can relax the muscles and stimulate the blood circulation and has been applied in clinical medicine. In the present study, we evaluated protective effect of ShuJinHuoXue tablets against IR-induced oxidative injury in skeletal muscle of experimental animals.

## 2. Results and Discussion

NO is a major mediator of tissue damage during ischemia reperfusion injury. NO displays dual cytoprotection and cytotoxicity actions, which makes elucidation of its exact role in biological systems difficult. Previous studies have demonstrated an increased production of nitric oxide (NO) in the brain during ischemia [18,19,20,21,22]. Examination of the role of NO in mediating the severity of skeletal muscle and cardiac IRI has led to some interesting and novel techniques. Administration of NO donors has helped elucidate the mechanisms and biochemical pathways involved in the pathogenesis of skeletal muscle IRI. Since NO produced by epithelial cells or cells in nearby tissue regulates peripheral circulation due to its vasodilating effect, NOS may be involved in the development of edema and tissue damage in muscle subjected to I/R. endothelin-1, a 21 amino acid peptide produced by endothelial cells, produces intense and long-lasting vasoconstriction [23,24,25]. When ET-1 binds to the receptor, it may promote release of intracellular Ca^2+^ by activating phospholipase C pathway, or promote extracellular Ca^2+^ influx into cells by activating receptor gated calcium channel and two hydrogen pyridine sensitive voltage dependent calcium channel. This resulted in intracellular Ca^2+^ increase and vasoconstrictor effect. 

Table 1 shows that the serum NO, ET-1, TXB_2_, 6-Keto-PGF_1α_ levels and TXB_2_/6-Keto-PGF_1α_ ratio were significantly higher in the IR group than in the NC group; whereas NO/ET-1 ratio was markedly lower (*p* < 0.05). The SJHXT pre-treatment (200, 300 and 400 mg/kg/day) markedly reduced serum ET-1, TXB_2_, levels and TXB_2_/6-Keto-PGF_1α_ ratio, and increased serum NO, 6-Keto-PGF_1α_ levels and NO/ET-1 ratio in the IR+SJHXT groups (*p* < 0.05) compared to IR group. The results indicated that SJHXT pre-treatment (200, 300 and 400 mg/kg/day) could effectively improve the balance between NO/ET-1 and 6-keto-PGF1α/TXB, and consequently inhibit excessive vasoconstriction and improve blood vessel microcirculation.

**Table 1:** Effect of SJHXT on serum NO, ET-1, TXB_2_, 6-Keto-PGF_1α_ levels, NO/ET-1 ratio and TXB_2_/6-Keto-PGF_1α_ ratio in control and experimental groups.

Group	NO (μmol/L)	ET-1 (ng/L)	NO/ET-1	TXB_2_ (ng/L)	6-Keto-PGF_1α_ (ng/L)	TXB_2_/ 6-Keto-PGF_1α_
NC	31.67 ± 2.99	79.53 ± 6.81	0.39 ± 0.03	201.43 ± 18.57	659.03 ± 57.77	0.316 ± 0.028
IR	50.82 ± 4.82 ^b^	275.83 ± 24.47 ^b^	0.18 ± 0.01 ^b^	517.39 ± 49.05 ^b^	871.62 ± 80.32 ^b^	0.594 ± 0.063 ^b^
IR+SJHXT (200 mg/kg/day)	59.09 ± 6.48	227.09 ± 22.85 ^d^	0.26 ± 0.02 ^c^	487.27 ± 44.13 ^c^	968.26 ± 90.49 ^d^	0.503 ± 0.047 ^c^
IR+SJHXT (300 mg/kg/day)	66.48 ± 6.03 ^d^	197.53 ± 21.68 ^d^	0.34 ± 0.03 ^d^	451.38 ± 48.01 ^d^	1083.44 ± 93.68 ^d^	0.416 ± 0.038 ^d^
IR+SJHXT (400 mg/kg/day)	71.24 ± 8.01 ^d^	152.69 ± 17.05 ^d^	0.47 ± 0.04 ^d^	406.14 ± 37.84 ^d^	1192.49 ± 121.07 ^d^	0.341 ± 0.033 ^d^

^b^ *p <*0.01, compared with NC group; ^c^ *p <* 0.05, ^d^ *p <* 0.01, compared with IR group; NC: normal control.

Normal skeletal muscle cells contain plenty of CK and LDH enzymes. The cytosolic enzyme CK is found predominantly in muscle and is a reliable marker of muscle tissue damage [26]. Lactate dehydrogenase is also a cytosolic enzyme found in the muscle, but is present in many other tissues as well [27]. Consequently, LDH was a less specific measure of muscle injury, but still a measure of general tissue injury.

In the present study, serum CK and LDH activities in IR group were statistically increased compared to NC group (Table 2). Pre-treatment of SJHXT (200, 300 and 400 mg/kg/day) significantly reversed the IR-induced increased activities of serum CK and LDH to normal in serum in the IR+SJHXT groups. Our work showed that SJHXT may effectively inhibit CK and LDH to release into blood, indicating that SJHXT can decrease ischemia-reperfusion induced oxidative injury in skeletal muscle.

**Table 2:** Effect of SJHXT on serum CK and LDH activities.

Group	CK (μkat/L)	LDH (μkat/L)
NC	93.06 ± 11.76	6.37 ± 0.77
IR	424.57 ± 52.87 ^b^	22.81 ± 2.41 ^b^
IR+SJHXT (200 mg/kg/day)	328.06 ± 47.18 ^d^	16.77 ± 2.05 ^d^
IR+SJHXT (300 mg/kg/day)	247.11 ± 36.01 ^d^	11.59 ± 1.31 ^d^
IR+SJHXT (400 mg/kg/day)	169.03 ± 18.09 ^d^	8.04 ± 0.92 ^d^

^b^ *p* < 0.01, compared with NC group; ^d^ *p* < 0.01, compared with IR group.

Increased myeloperoxidase (MPO) activity after reperfusion of ischemic muscle demonstrates an influx of neutrophils in muscle tissue during reperfusion [28,29,30], and the extravasated neutrophils play an active role in ischemia-reperfusion injury [31,32,33,34]. Table 3 showed that serum MPO activity and wet weight/dried weight (W/D) ratio in IR group were significantly higher than those in NC group. In the IR+SJHXT groups, pre-treatment of SJHXT (200, 300 and 400 mg/kg/day) significantly decreased serum MPO activity and W/D ratio in a dose-dependent manner compared to IR group. This suggested that SJHXT may effectively decrease neutrophils-induced tissue inflammatory infiltration. In addition, decreased W/D ratio in IR + SJHXT groups indicated that SJHXT pre-treatment can decrease IR-induced skeletal muscle tissue edema.

**Table 3:** Effect of SJHXT on serum MPO activity and W/D ratio.

Group	MPO (μkat/L)	W/D
NC	1.46 ± 0.18	5.01 ± 0.48
IR	4.39 ± 0.51 ^b^	6.94 ± 0.66 ^b^
IR+SJHXT (200 mg/kg/day)	3.88 ± 0.42 ^c^	6.43 ± 0.59
IR+SJHXT (300 mg/kg/day)	3.05 ± 0.29 ^d^	5.92 ± 0.55 ^c^
IR+SJHXT (400 mg/kg/day)	2.41 ± 0.22 ^d^	5.61 ± 0.48 ^c^

^b^ *p* < 0.05, compared with NC group; ^c^ *p* < 0.05, ^d^ *p* < 0.05, compared with IR group.

Previous studies have shown that tissue IRI was closely associated with rapid free radical production and intracellular Ca^2+^ accumulation [35]. It is well known that Na^+^-K^+^ ATPase is involved in the cleavage of ATP to release Pi [36]. Na^+^-K^+^ ATPase is dominantly expressed in skeletal muscles [37] and is involved in the transport of Na^+^ and K^+^ in the membrane. The Na^+^, K^+^-ATPase forms an integral part of the Na^+^, K^+^ pump and the splitting of ATP provides the energy required to drive the active transport of the cations. The (Ca^2+^-Mg^2+^) ATPase (ATPase) from skeletal muscle sarcoplasmic reticulum is one of most studied membrane proteins. The ATPase couples the transport of 2 moles of Ca^2+^ across the sarcoplasmic reticulum membrane with the hydrolysis of 1 mol of ATP. A conformational change accompanies the reaction cycle [38,39].

Table 4 shows that skeletal muscle Ca^2+^ level in the IR group was significantly higher, whereas Na^+^-K^+^-ATPase and Ca^2+^-Mg^2+^-ATPase activities were markedly lower than those in the NC group. In the IR+SJHXT groups, pre-treatment of SJHXT (200, 300 and 400 mg/kg/day) significantly decreased skeletal muscle Ca^2+^ levels and increased skeletal muscle Na^+^-K^+^-ATPase and Ca^2+^-Mg^2+^-ATPase activities in a dose-dependent manner compared to IR group.

**Table 4:** Effect of SJHXT on skeletal muscle Ca^2+^ level, Na^+^-K^+^-ATPase and Ca^2+^-Mg^2+^-ATPase activities.

Group	Ca^2+^ (mmol/g prot)	Na^+^-K^+^-ATPase (μmol Pi/mg prot/hour)	Ca^2+^-Mg^2+^-ATPase (μmol Pi/mg prot/hour)
NC	0.062 ± 0.007	2.38 ± 0.27	2.57 ± 0.31
IR	0.191 ± 0.013 ^b^	1.27 ± 0.14 ^b^	1.39 ± 0.15 ^b^
IR+SJHXT (200 mg/kg/day)	0.145 ± 0.011 ^c^	1.61 ± 0.19	1.75 ± 0.19 ^c^
IR+SJHXT (300 mg/kg/day)	0.107 ± 0.012 ^d^	1.93 ± 0.21 ^c^	2.28 ± 0.25 ^d^
IR+SJHXT (400 mg/kg/day)	0.082 ± 0.007 ^d^	2.22 ± 0.24 ^d^	2.49 ± 0.26 ^d^

^b^ *p* < 0.05, compared with NC group; ^c^ *p* < 0.05, ^d^ *p* < 0.05, compared with IR group.

Oxidative stress has been described as a disturbance in the equilibrium status of pro-oxidant/antioxidant systems in intact cells. Thus, ROS have a number of features which include impaired muscle contractions, muscle necrosis, endothelial cell swelling, release of cellular proteins and increased microvascular permeability to proteins [40,41]. Since ROS during ischemia and reperfusion is supposed to play a major role, several antioxidants like N-acetylcysteine, vasoactive intestinal peptide, SOD, iloprost, and vitamin E have been used to reverse skeletal muscle I/R damage [42,43]. ROS during ischemia and reperfusion to the skeletal muscle have a number of features, which include impaired muscle contractions, muscle necrosis, endothelial cell swelling, release of cellular proteins, and increased microvascular permeability to proteins [44,45,46].

MDA level in skeletal muscle of IR group was significantly increased in that in NC group (Table 5). In the IR+SJHXT groups, pre-treatment of SJHXT (200, 300 and 400 mg/kg/day) significantly decreased skeletal muscle MDA level in a dose-dependent manner compared to IR group.

**Table 5:** Effect of SJHXT on skeletal muscle MDA level, SOD, CAT and GSH-Px activities.

Group	MDA (nmol/mg protein)	SOD (U/mg protein)	CAT (U/mg protein)	GSH-Px (U/mg protein)
NC	3.16 ± 0.34	304.7 ± 34.15	68.31 ± 7.16	73.09 ± 6.88
IR	7.92 ± 0.68 ^b^	168.3 ± 18.59 ^b^	35.05 ± 4.03 ^b^	30.61 ± 3.71 ^b^
IR+SJHXT (200 mg/kg/day)	6.24 ± 0.66 ^d^	199.4 ± 22.11 ^d^	47.41 ± 5.12 ^d^	48.92 ± 5.05 ^d^
IR+SJHXT (300 mg/kg/day)	5.02 ± 0.57 ^d^	264.8 ± 30.08 ^d^	57.07 ± 6.06 ^d^	60.51 ± 7.82 ^d^
IR+SJHXT (400 mg/kg/day)	4.28 ± 0.51 ^d^	290.4 ± 31.49 ^d^	66.17 ± 7.29 ^d^	79.03 ± 6.79 ^d^

^b^ *p* < 0.05, compared with NC group; ^d^ *p* < 0.05, compared with IR group.

In addition, pre-treatment of SJHXT (200, 300 and 400 mg/kg/day) significantly reversed the IR-induced antioxidant enzymes activities in skeletal muscle of the IR+SJHXT groups compared to IR group. In the present study, increased skeletal muscle MDA, Ca^2+^ levels, decreased Na^+^-K^+^-ATPase, Ca^2+^-Mg^2+^-ATPase and antioxidant enzymes (SOD, CAT and GSH-Px) activities suggested that IR may lead to excessive free radicals production, increased lipid peroxidation level and Ca^2+^ overload. Pre-treatment of SJHXT (200, 300 and 400 mg/kg/day) may decrease skeletal muscle lipid peroxidation level and increase antioxidant enzymes activities, and consequently reduce Ca^2+^ overload. As a result, SJHXT pretreatment decreased free radicals induced vascular endothelial permeability and ischemic tissue edema and necrosis.

**Figure 1:**
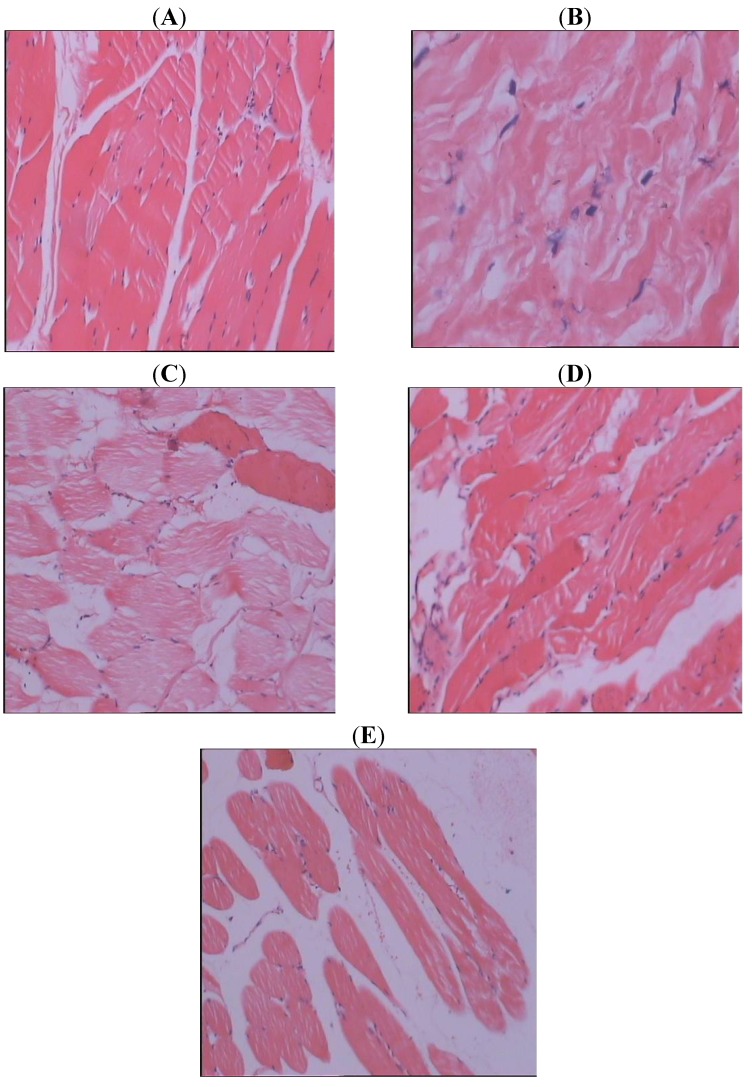
H&E staining **A**: NC; **B**: IR; **C**: IR+SJHXT (200 mg/kg/day); **D**: IR+SJHXT (300 mg/kg/day); **E**: IR+SJHXT (400 mg/kg/day).

In the NC group, regular muscle fiber morphology was observed, and staining was uniform (Figure 1). In the IR group, irregular muscle fiber morphology was observed, and muscle fiber structure was disordered, and shrinkage was observed, and cell gaps were obviously widened. In IR+SJHXT groups, muscle fiber morphology was more regular, and staining was uniform, most of the muscle cell was still located in the muscle membrane under clings to the muscle fibers of the peripheral, muscle fiber swelled, cacuolar degeneration was not obvious, and cell gap slightly wide.

## 3. Experimental

### 3.1. Materials

ShuJinHuoXue tablets were purchased from Sanmenxia Xinyuan Pharmaceutical Co. Ltd (Sanmenxia, China).

### 3.2. Animals

Rabbits were obtained from the Laboratory Animal Institute of our hospital (Dalian, China). Animals were kept in an environmentally controlled breeding room (temperature: 20 ± 2 °C, humidity: 60 ± 5%, 12 h dark/light cycle) for 1 week before the start of the experiments. They were fed standard laboratory chow with water *ad libitum* and fasted overnight before the experiments. Experimental animals were maintained in accordance with internationally accepted principles for laboratory animal use. Animals were divided into five groups [normal control (NC) group, ischemia reperfusion (IR) group, three doses of SJHXT-treatment (IR + SJHXT) groups]. Each group contained 10 animals. Animals in the NC group and IR group received daily gastric gavage with distilled water for 40 days. The other three SJHXT-treatment groups received 200, 300 and 400 mg/kg/day p.o. ShuJinHuoXue tablets for 40 days; appropriate doses were identified using our pre-experimental work.

All surgical procedures were performed while the rabbits (IR group, IR + SJHXT groups) were under anesthesia with intraperitoneally administered 60 mg kg^−1^ ketamine and 10 mg kg^−1^ xylazine cocktail. A skin incision was made over the anteromedial surface of the right hind limb, starting at the level of femoral artery, extending upward to the inguinal ligament. The right femoral artery was isolated by clamping with an atraumatic microvascular clamp. After the animals were anesthetized, an ischemic insult was created in the right femoral artery for 2 h, followed by 4 h of reperfusion.

Serum was collected from whole blood for biochemical analysis. Skeletal muscle was harvested from injured and control hind limbs; the tissue was either fixed for histologic evaluation or snap-frozen in liquid nitrogen and then stored at −80 °C for biochemical analysis. 

### 3.3. Biochemical Assays

Serum nitric oxide levels were determined by using an enzyme-linked immunosorbent assay (ELISA) test kit purchased from NanJing JianChen Biotechnology Ltd (Nanjing, China). Serum lactate dehrdrogenase (LDH) and creatine kinase (CK) were determined using kits also purchased from NanJing JianChen Biotechnology Ltd. The level of Endothelin-1 (ET-1) in rabbit plasma was determined using an ELISA kit (Y-Y Chemical Reagent Co., Ltd). The optical density at the wavelength of 450 nm was measured using an EIX-800 intrument from BIO-TEK INSTRUMENTS Company (Winooski, VT, USA) in order to calculate the level of ET-1. Ca^2+^ levels were measured using a Model 634 Ionized Ca Analyzer (Ciba Corning Diagnostics, Medford, MA, USA). MPO activity was assessed by measuring the hydrogen-peroxide-dependent oxidation of *o*-dianisidine dihydrochloride. One enzyme unit was defined as the amount of enzyme producing one absorbance change per minute at 460 nm and 37 °C [47]. 

Activities of Na^+^/K^+^-ATPase and Ca^2+^-Mg^2+^-ATPase from skeletal muscle were determined by the method of Gerbi *et al*. [48] and Yoshioka and Tanaka [49], respectively. The activities were indirectly measured by estimating the phosphorous liberated after the incubation of cardiac tissue homogenate in a reaction mixture containing the substrate ATP with the co-substrate elements at 37 °C for 15 min. The reactions were arrested by adding 1.0 mL of 10% trichloroacetic acid (TCA). The phosphorus content from the TCA supernatants was then determined by the method of Fiske and Subbarow [50]. ATPases activity expressed as μmol of phosphorus liberated/mg protein/hour at 37 °C.

The tissue MDA concentration was determined using the method described by Jain *et al*. [51], based on TBA reactivity. Briefly, supernatant obtained from tissues (0.2 mL), phosphate buffer (pH 7.4, 0.8 mL), BHT (0.025 mL) and 30% TCA (0.5 mL) were added to the tubes and mixed. After 2 h incubation at −20 °C, the mixture was centrifuged (4000 × g) for 15 min. After this, supernatant (1 mL) was taken and added to each tube, and then 0.1M EDTA (0.075 mL) and 1% TBA (0.25 mL) were added. These tubes with Teflon-lined screw caps were incubated at 90 °C in a water bath for 15 min and cooled to room temperature. The optical density was measured at 532 nm for tissue MDA concentration (Novaspec II Pharmacia-Biotech, Biochrom Ltd., Cambridge, UK).

SOD activity assay. Skeletal muscle tissue was ground in liquid nitrogen and suspended in a homogenization buffer consisting of 50 mM Tris-HCl, pH 8.2, 1 mM EDTA, 0.1% Triton X-100, and proteinase inhibitor cocktail (Roche, Mannheim, Germany). After centrifugation in a microcentrifuge at 4 °C, the supernatants were used to determine enzyme activity and protein concentration. The SOD activity was measured spectrophotometrically using the method developed by Marklund and Marklund [52]. Briefly, SOD was detected on the basis of its ability to inhibit superoxide-mediated reduction. One unit was determined as the amount of enzyme that inhibited oxidation of pyrogallol by 50%. 

The activity of catalase was measured according to the method of Aebi [53]. The reaction mixture (1 mL) that contained phosphate buffer (0.1 M, pH 7.4, 0.78 mL), liver supernatant (0.2 mL), and H_2_O_2_ (0.5 M, 0.02 mL) was prepared. The reaction was started by adding H_2_O_2_ and decomposition was monitored by following the decrease in absorbance at 240 nm for 1 min. 

Activity of glutathione peroxidase (GSH-Px) was determined according to the method of Lawrence and Burk [54]. The assay mixture consisted of 75 mM phosphate buffer (pH 7.0, 2.0 mL), 60 mM glutathione (50 μL), 30 units/mL glutathione reductase (0.1 mL), 15 mM EDTA (0.1 mL), 3 mM NADPH (0.1 mL) and the appropriate amount of tissue supernatant to a final volume of 3.0 mL. The reaction was started by the addition of 7.5 mM H_2_O_2_ (0.1 mL). The rate of change of absorbance during the conversion of NADPH to NADP^+^ was recorded spectrophotometrically at 340 nm for 3 min. 

### 3.4. Wet Weight/Dried Weight Assay

After muscle function was assessed, the limb muscles were excised and weighed (wet weight). The muscles were then dried at 60 °C in a convection oven for 72 h and reweighed (dry weight). The resulting W/D ratios were used as indices of edema formation.

### 3.5. Histopathological Study

Skeletal muscle samples were dissected and fixed in 10% neutral formalin, dehydrated in ascending grades of alcohol and imbedded in paraffin wax. Paraffin sections (5 μm thick) were stained for routine histological study using haematoxylin and eosin (H&E).

### 3.6. Statistics

All data are presented as means ± SE. The results were calculated statistically using 1-way analysis of variance (ANOVA) and the Duncan multiple range test. Differences were considered to be significant at *p* < 0.05. Data were evaluated using the Sigma Stat (version 13.0) [55] statistical analysis program (SPSS Inc., Chicago, IL, USA).

## 4. Conclusion

SJHXT pre-treatment may inhibit excessive contraction of a blood vessel by regulating between NO/ET-1 and 6-keto-PGF1A/TXB2, improve skeletal muscle blood vessel microcirculation, decrease skeletal muscle tissue Ca^2+^ overload and oxidative injury in skeletal muscle IR rabbits. Moreover, histopathological study further confirms that SJHXT pre-treatment can alleviate IR-induced skeletal muscle tissue injury in rabbits. As a potent antioxidative and anti-inflammatory agent in pharmacologically applicable doses, SJHXT may be beneficial for the prevention and/or amelioration of IR-induced skeletal muscle injury.
